# No significant regulation of *bicoid* mRNA by Pumilio or Nanos in the early *Drosophila* embryo

**DOI:** 10.1371/journal.pone.0194865

**Published:** 2018-03-30

**Authors:** Tammy H. Wharton, Krystle J. Nomie, Robin P. Wharton

**Affiliations:** 1 Departments of Molecular Genetics & Cancer Biology and Genetics, Center for RNA Biology Ohio State University Columbus, OH; 2 Department of Lymphoma/Myeloma MD Anderson Cancer Center Holcombe Blvd. Houston, TX; East Carolina University, UNITED STATES

## Abstract

Drosophila Pumilio (Pum) is a founding member of the conserved Puf domain class of RNA-binding translational regulators. Pum binds with high specificity, contacting eight nucleotides, one with each of the repeats in its RNA-binding domain. In general, Pum is thought to block translation in collaboration with Nanos (Nos), which exhibits no binding specificity in isolation but is recruited jointly to regulatory sequences containing a Pum binding site in the 3’-UTRs of target mRNAs. Unlike Pum, which is ubiquitous in the early embryo, Nos is tightly restricted to the posterior, ensuring that repression of its best-characterized target, maternal *hunchback* (*hb*) mRNA, takes place exclusively in the posterior. An exceptional case of Nos-independent regulation by Pum has been described—repression of maternal *bicoid* (*bcd*) mRNA at the anterior pole of the early embryo, dependent on both Pum and conserved Pum binding sites in the 3’-UTR of the mRNA. We have re-investigated regulation of *bcd* in the early embryo; our experiments reveal no evidence of a role for Pum or its conserved binding sites in regulation of the perdurance of *bcd* mRNA or protein. Instead, we find that Pum and Nos control the accumulation of *bcd* mRNA in testes.

## Introduction

Post-transcriptional mechanisms play a preeminent role in the initial steps of the regulatory cascade that governs antero-posterior development in the Drosophila embryo. Anterior segmentation is controlled by Bcd [1, 2], which is translated from maternally synthesized *bcd* mRNA that is localized to the anterior cortex [3]; after fertilization the mRNA is released from the poorly understood process of masking that inhibits its translation during oogenesis. Posterior segmentation is controlled by Nos [4], which is translated at the end of oogenesis from the subset of maternally synthesized *nos* mRNA that is localized to the pole plasm at the posterior cortex [5]; the remaining *nos* mRNA (~96% of the total *nos* mRNA) is distributed uniformly throughout the prospective somatic cytoplasm and repressed to ensure that Nos is generated only transiently at the posterior pole [6].

Nos itself is a repressor that regulates segmentation by blocking translation of maternal *hb* mRNA [7–9]. In addition to this function in early embryonic development, Nos plays many other roles in the organism, including regulation of several aspects of the development of primordial germ cells (PGCs) [10–12], maintenance of the stem cell fate in the ovarian germ line [13], regulation of several aspects of neuronal development and activity [14–16], and buffering cells against the loss of Retinoblastoma (Rb) activity [17]. Studies of mammalian cells and mice have revealed that the Rb-buffering and germ line cell maintenance functions of Nos are evolutionarily conserved [17, 18].

Nos does not appear to recognize target mRNAs on its own; experiments on three well-studied Nos regulatory targets—*hb*, *bcd*, and *CycB*—have shown that Nos binds jointly with Pumilio (Pum) to conserved sequence elements in each 3’-UTR [19–21]. Pum is a high-specificity RNA-binding protein that is uniformly distributed throughout the early embryo [22]. Pum recognizes 8 nucleotides, one with each of the 8 repeats that constitute its RNA-binding Puf domain [23]. In contrast, Nos has essentially no binding specificity of its own [21, 24], but can be recruited specifically to Pum-RNA complexes [20, 21]. Recent structural studies have shown that the C-terminal tail of Nos interacts with the Puf RNA-binding domain to alter its conformation, allowing the two proteins to bind cooperatively to sites that function in vivo as Nanos Regulatory Elements (NREs) [21, 25].

Pum binds with high affinity to NRE-containing target mRNAs in the absence of Nos, raising the possibility that Pum might have Nos-independent regulatory effects on a subset of its targets. Two main lines of evidence support such an idea. The first comes from studies of budding yeast homologues that have the conserved Pum-and-FBF (PUF) RNA-binding domain. Budding yeast has no Nos homologue; a number of studies have shown that yeast PUF proteins bind on their own to target mRNAs and recruit effectors that regulate translation and mRNA stability via direct protein-protein interactions [26, 27]. The second line of evidence comes from study of the regulation of *bcd* mRNA at the anterior of the embryo, where Nos activity is undetectable.

Although Nos can collaborate with Pum to repress *bcd* translation via the NRE, in wild type embryos it does not; the ability of Nos to repress *bcd* is revealed only upon mis-expression of Nos at the anterior of the embryo [28, 29]. However, Gamberi et al. [30] found that accumulation of maternal *bcd* mRNA and protein is significantly prolonged in embryos from *pum* mutant females. In addition, they found that mutations in the Pum binding sites within the *bcd* NRE caused gross defects in head development and organismal lethality. Taken together, these results have been quoted as evidence of Nos-independent regulation by Pum in vivo.

In this report we investigate the mechanistic similarity between Drosophila Pum and the yeast PUF proteins, re-examining potential Nos-independent regulation by Pum in the early embryo. We find that neither Pum nor Nos significantly governs the perdurance of *bcd* gene products in the early embryo. Instead, Pum and Nos appear to modulate *bcd* mRNA levels in the testes.

## Results

### Normal persistence of *bcd* mRNA and protein in *pum* and *nos* mutant embryos

In the course of examining the regulation of maternal mRNAs in vivo, we monitored *bcd* mRNA persistence in wild type and *pum* mutant embryos. To more precisely age embryos during the critical stage of development when many maternal mRNAs are degraded, we simultaneously detected *bcd* and *even-skipped* (*eve*) mRNAs by in situ hybridization. The pattern of *eve* expression evolves rapidly during stages 4–5 and has been carefully documented as part of a larger study of pair-rule gene expression [31].

**Fig 1:**
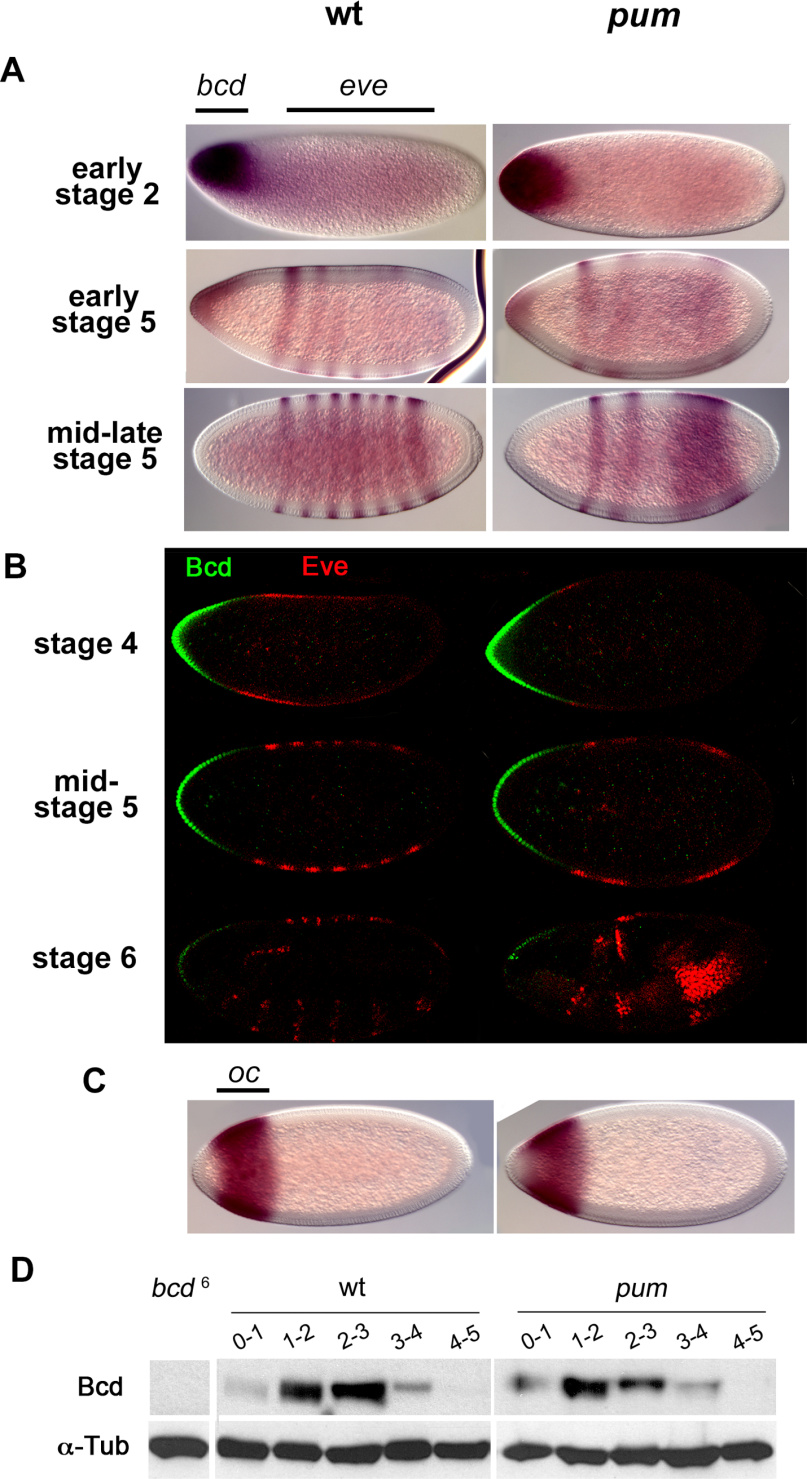
The timecourse of *bcd* mRNA and protein accumulation during early embryogenesis. Samples of embryos from wild type (wt) (left column) and *pum* null mutant females (right). (A) *bcd* and *eve* mRNAs visualized by in situ hybridization; the former is transcribed maternally and accumulates at the anterior, while the latter is transcribed zygotically and accumulates in seven stripes (in wt embryos) approximately in the regions of the embryos labeled above. *pum* mutant embryos lack posterior patterning activity and so the pattern of *eve* stripes 3–7 is altered. Approximate embryonic ages in hours post-fertilization are 0.75 (stage 2), 2.25 (early stage 5), 2.6 (mid-late stage 5). (B) Bcd (green) and Eve (red) proteins visualized by immunohistochemistry and confocal microscopy. Approximate embryonic ages are 1.75 hours (stage 4), 2.6 hours (mid-stage 5), and 3 hours (stage 6). Note that only a trace of Bcd is detectable at the onset of gastrulation (stage 6) under these conditions, but low levels of residual protein are readily visible at increased gain. (C) *ocelliless* (*oc*) mRNA; *oc* is a direct Bcd transcriptional target. (D) Timecourse of Bcd accumulation as revealed by Western blots, of hand-sorted samples, with embryonic age in hours above. The left lane is a negative control of 0–3 hour embryos from *bcd*^6^ females that produce no stable Bcd. As a loading control, the membrane was re-probed with an antibody to alpha-tubulin (below).

To our surprise, *bcd* mRNA was degraded with a normal time course in the absence of Pum function: the mRNA was abundant in embryonic stages 2–4, and degraded early in stage 5 such that by mid stage 5 essentially no detectable *bcd* mRNA remains (Fig 1A).

As Pum primarily acts to repress translation rather than promote degradation of target mRNAs, we next asked whether Pum might regulate the accumulation of *bcd* protein. As shown in Fig 1B, high levels of Bcd are present in a gradient at the anterior of both wild type and *pum* mutant embryos at stage 4. During stage 5, the level of Bcd declines until it is barely detectable at the onset of gastrulation; throughout early development, the level of Bcd protein in wild type and *pum* mutant embryos is essentially indistinguishable. Finally, if Bcd persisted longer in the absence of Pum activity, then direct targets of Bcd might accumulate to higher levels or persist later into development. However, we observe no difference in the accumulation of one such target, *ocellilis* (*oc*) mRNA [32], in the absence of Pum function (Fig 1C and not shown).

The results described above are at odds with previous work, in which essentially the same (high) level of *bcd* mRNA was observed in samples of *pum* mutant embryos (but not wild type embryos) that correspond to stages 2 and 5 [30]. In addition, high levels of *bcd* protein were observed in *pum* mutant embryos in samples that correspond to gastrulating embryos (stage 6) [30]. What might account for the difference between these studies and our observations in Fig 1? In the previous studies, *bcd* gene products were monitored not in individual embryos, whose developmental stage can be readily assessed, but in homogenized samples prepared from pools of embryos; these were subsequently analyzed by Northern or Western blot to detect mRNA and protein, respectively. We examined carefully aged collections of embryos and found they contain variable proportions of unfertilized eggs. Since *bcd* mRNA and protein persist in unfertilized eggs [2, 33], their inadvertent inclusion in pooled samples would artefactually increase the apparent amount of *bcd* gene products in pools comprised primarily of stage 5 (or older) embryos.

Consistent with this idea, when we initially measured Bcd protein levels in timed collections by Western blot, results were extremely variable; but high levels of protein were usually observed in samples nominally comprised of wild type stage 5 (or older) embryos (not shown). To eliminate the contribution of “inappropriately aged” embryos in each collection, we fixed embryos with methanol, stained nuclei with DAPI, and manually sorted each pool [34] to exclude unfertilized eggs as well as older embryos (common when females hold fertilized eggs before depositing them) before preparing the remainder for analysis by Western blot. As shown in Fig 1D, using this approach we see no significant effect on Bcd protein persistence upon elimination of Pum activity—only a low level of Bcd is detected from 3–4 hours of development (corresponding to stages 6–8) and only a trace of protein is detectable subsequently.

**Fig 2:**
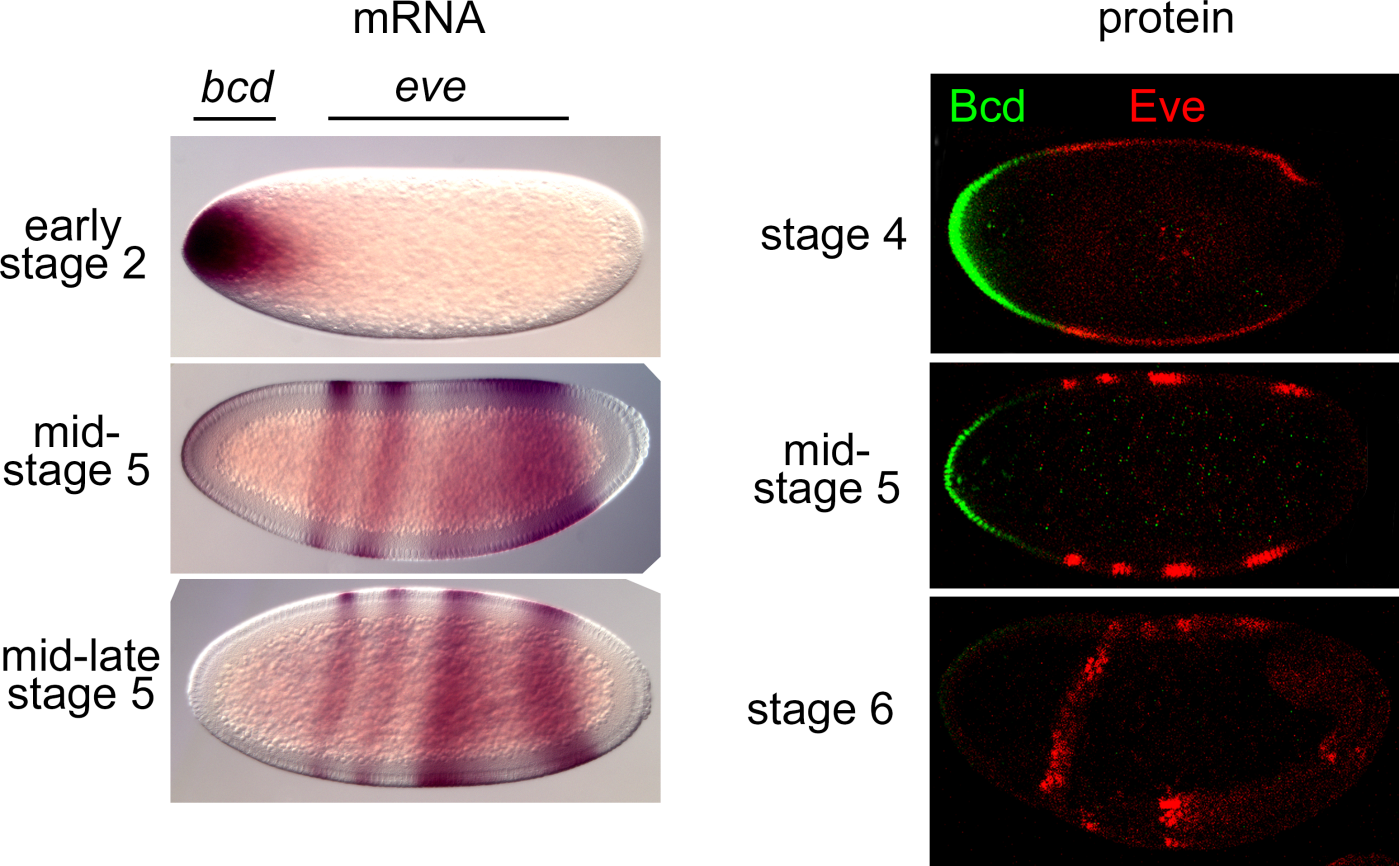
The timecourse of *bcd* mRNA and protein accumulation in the absence of Nos activity. *bcd* and *eve* mRNAs (left) and proteins (right) in embryos with no detectable *nos* activity, detected as described in Fig 1.

Although *bcd* mRNA is not thought to be exposed to meaningful levels of Nos in wild type embryos, we nevertheless repeated a subset of the experiments described above to ask whether loss of Nos activity might indirectly affect the timecourse of *bcd* mRNA or protein disappearance. As shown in Fig 2, we see no evidence for a significant effect of Nos: *bcd* mRNA is degraded during stage 5 on schedule and Bcd protein is reduced to very low levels by the onset of gastrulation in *nos* mutant embryos.

We conclude that neither Pum nor Nos exerts an appreciable effect on the amount or persistence of *bcd* mRNA or protein during early embryogenesis.

### Normal development upon ablation of the *bcd* NRE

While most of the maternally synthesized *bcd* mRNA is degraded by mid- to late-stage 5 (Fig 1), a low residual level of *bcd* mRNA (undetectable by the in situ hybridization methods we employed) persists throughout embryonic development, as seen in high-throughput sequencing experiments [35]. Although we see no evidence of Nos-independent regulation by Pum during stages 1–5 of embryogenesis (Fig 1), in theory the “residual” low level of *bcd* mRNA might be subject to regulation during later stages of embryonic development. Such an idea is consistent with the claim that up to 89% of embryos from transgenic females expressing a Pum-resistant mutant *bcd* mRNA exhibit readily visible head defects [30], similar to the lethal phenotypes in embryos lacking apoptosis effectors [36]. No such dominant defects were observed in earlier studies of embryos bearing a Pum-resistant maternal *bcd* NRE(Δ) mRNA [25], but many properties of the transgenic flies were different in these studies. To resolve the apparent discrepancy, we re-investigated the possibility that the *bcd* NRE plays a role in post-gastrulation embryonic development as follows.

**Fig 3:**
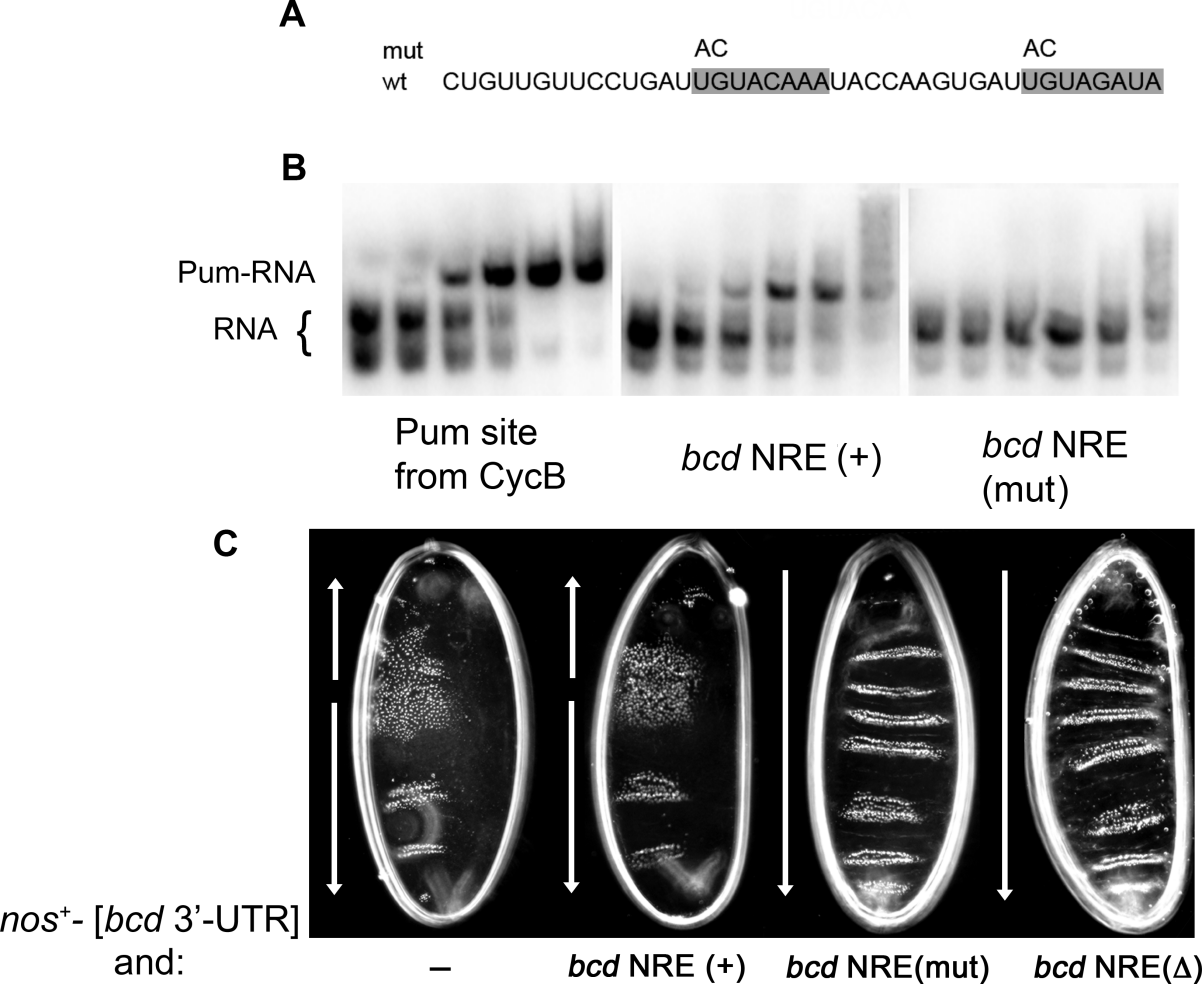
Mutations in the *bcd* NRE abolish binding of Pum in vitro and in vivo. (A) Sequence of the 45 nt wt *bcd* NRE, with the 4 substitutions in the mutant (mut) NRE above. Canonical Pum binding sites in shaded boxes. (B) Gel mobility shift experiments to assay Pum binding. An increasing concentration of the Pum RNA-binding domain (0, 0.25, 0.5, 1.0, 2.0, 4.0 μM in lanes 1–6 of each panel) was incubated with the RNAs indicated below and electrophoresed to separate bound and free RNA. The Pum binding site in the *CycB* NRE [19] is a positive control. (C) An in vivo assay for NRE-dependent regulation of *bcd* mRNA in embryos that have ectopic Nos at the anterior by virtue of maternal expression of a chimeric mRNA that is localized to the anterior pole of the embryo by its 3’-UTR (from *bcd*) and encodes wild type *nos* protein. Dark field micrographs reveal primarily the pattern of abdominal segmentation; arrows indicate segmental and embryonic polarity. All embryos were from females bearing the *nos*^*+*^*—*[*bcd* 3’-UTR] transgene that results in the accumulation of ectopic anterior Nos; in addition, the females either had no additional transgene (-) or a single copy of a wild type *bcd* NRE (+), *bcd* NRE(mut), or *bcd* NRE(Δ) transgene, as indicated.

We first introduced point mutations into the *bcd* NRE (Fig 3A) and tested whether these compromise binding to Pum in vitro. As shown in Fig 3B, while the wild type *bcd* NRE binds Pum with an affinity similar to that of the *CycB* NRE in gel mobility shift experiments, the mutant NRE does not. We then generated new transgenic flies encoding *bcd* NRE(mut), as well as *bcd* NRE(+) and mutant *bcd* NRE(Δ) as negative and positive controls, respectively. We used transgenic lines that express normal levels of gene product by the criterion that they rescue the maternal effect on embryonic patterning of *bcd*^6^ homozygotes. We then showed that the mutant NRE does not bind Pum in vivo using the following assay. Ectopic anterior Nos (encoded by a *nos*^+^—[*bcd* 3’-UTR] transgene [28]) blocks translation of wild type *bcd* mRNA via its NRE, which (in part) causes development of a bicaudal embryonic body plan, with characteristic polarity reversal and the transformation of anterior segments into posterior ones. As described previously [25], expression of *bcd* NRE(Δ) mRNA suppresses the bicaudal phenotype, since the mRNA cannot be regulated by Nos+Pum. We find that expression of *bcd* NRE(mut) mRNA suppresses the bicaudal phenotype associated with ectopic anterior Nos in a similar manner, whereas expression of wild type *bcd* (NRE+) mRNA does not (Fig 3C). In summary, the mutant NRE in these experiments does not bind Pum to a significant extent either in vitro or in vivo.

**Fig 4:**
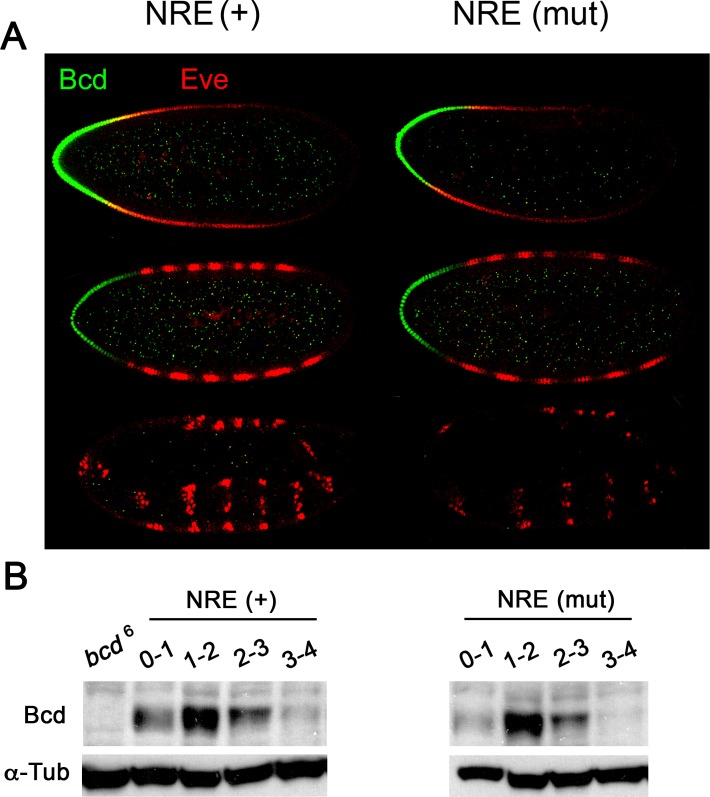
Bcd accumulation in embryos bearing a Pum-resistant *bcd* NRE (mut) mRNA. (A) Bcd (green) and Eve (red) proteins in embryos from otherwise wild type females that bear a single copy of a *bcd* transgene with a wild type (+) (left) or mutant (mut) NRE (right). Embryonic stages as in Fig 1B. (B) Western blot of sorted samples (developmental age in hours post-fertilization above) of embryos bearing mRNA from either a *bcd* NRE(+) control transgene or the *bcd* NRE (mut) transgene, as indicated. On the left, the negative control sample of *bcd*^6^ mutant embryos reveals two proteins that cross-react with the anti-Bcd antibody, one of which serves as a convenient loading control for the blot. The left lane is a negative control of 0–3 hour embryos from *bcd*^6^ females that produce no stable Bcd. As a loading control, the membrane was re-probed with an antibody to alpha-tubulin (below). Note that the *bcd* transgenes encode wild type protein and therefore the Bcd detected on the blot is derived from both the endogenous *bcd*^+^ genes and the transgene.

We next determined whether the Pum-resistant *bcd* NRE(mut) mRNA is mis-regulated during stages 1–6 of embryonic development. In embryos from otherwise wild type females that also bear a transgene encoding either *bcd* (NRE+) or *bcd* NRE(mut) mRNA, Bcd protein disappeared on schedule during stage 5 (Fig 4A). In addition, timed collections and analysis by Western blot of sorted embryos from transgenic *bcd* (NRE+) and *bcd* NRE(mut) females reveals that Bcd falls to very low levels on schedule, after 3 hours of embryonic development (Fig 4B). Note that the experiments of Fig 4 detect the wild type Bcd protein encoded by the endogenous *bcd*+ genes and the relevant transgene. Taken together, the results described above show no significant effect of inactivating the NRE on perdurance of Bcd in early embryos.

**Fig 5:**
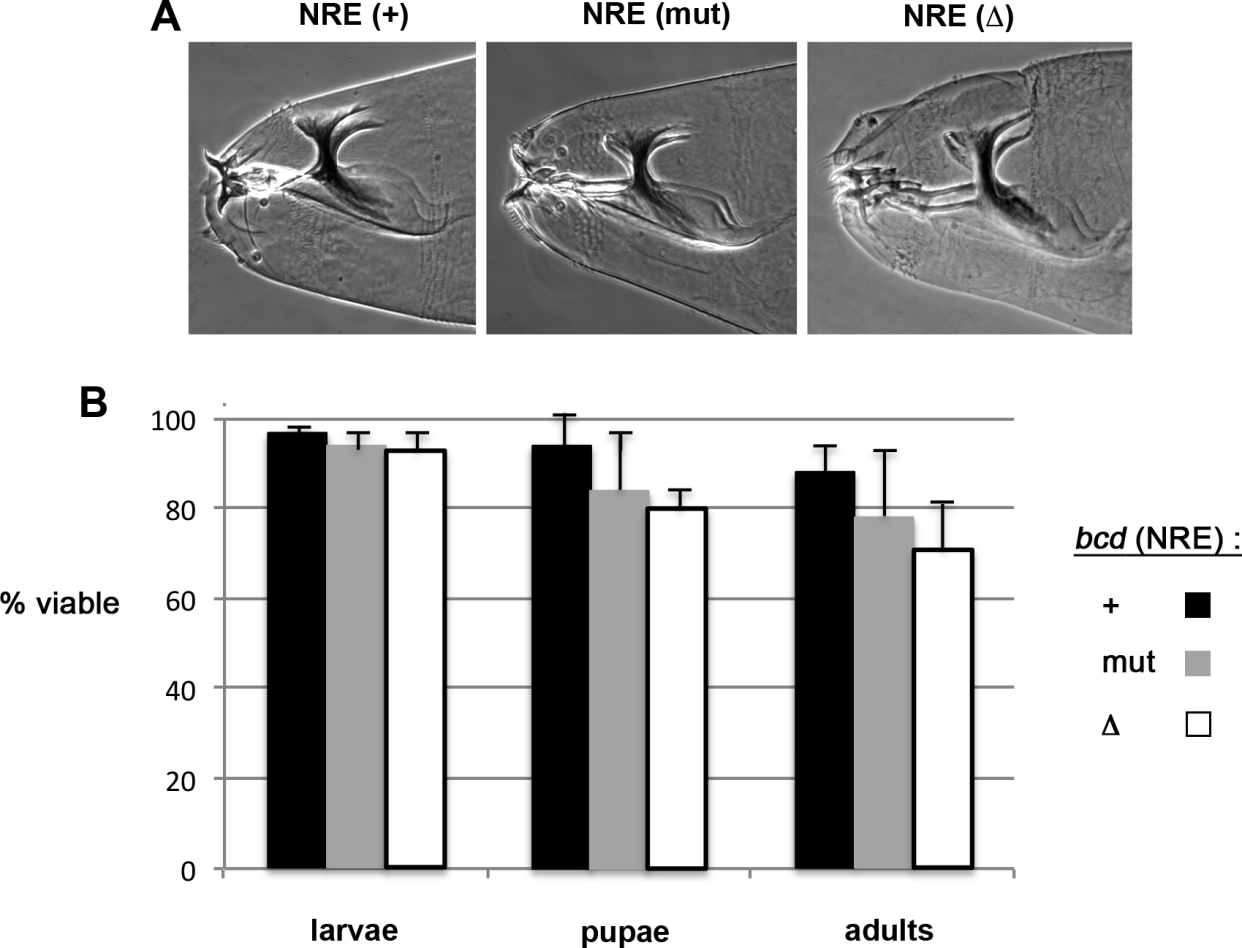
Viability of progeny developing from embryos with extra *bcd* mRNA. (A) Phase contrast micrographs of representative head skeletons of embryos derived from females bearing two copies of the *bcd* transgenes indicated above. (B) Percentage of viable animals at three different developmental stages (labeled below and to the right) that arise from embryos of “two-copy” females bearing *bcd* NRE(+), NRE(mut), or NRE(Δ) transgenes, as indicated. Each entry is the average for three different trans-heterozygous pairs of transgenes, with error bars showing the S.D. The figure reports the percentage of fertilized eggs that hatch into viable larvae, pupate, and eclose to viable adulthood. With one exception, at each stage of development there is no significant difference among all pairwise comparisons by two-tailed t-test; the exception is a modestly significant (P = 0.04) difference in pupal viability between NRE(+) and NRE(Δ) animals.

To examine a potential role for the *bcd* NRE in head development post-gastrulation, we monitored the development of embryos from trans-heterozygous females, each bearing two copies of an independently-derived *bcd* NRE(+), *bcd* NRE(mut), or *bcd* NRE(Δ) transgene. To minimize the probability that the site of transgene insertion might affect development, we prepared three different trans-heterozygous “two-copy” females for each transgene. Examination of cuticle preparations revealed that essentially all embryos from any of the two-copy females have normal head morphology (Fig 5A). We next followed the post-embryonic development of these animals, tracking the proportion of fertilized eggs that yield hatching larvae, pupae, and adults. As shown in Fig 5B, we observe no significant effect of mutating the NRE on development, with one minor exception. Viability to pupation is slightly reduced for animals from two-copy NRE(Δ) females (P = 0.04). This small reduction in viability is consistent with our observation that lines bearing a single NRE(Δ) transgene are slightly unhealthy. However, the main conclusion from these experiments is that embryos from females expressing the Pum-resistant *bcd* NRE(mut) mRNA exhibit normal head morphology and no significant reduction in viability. We cannot account for the discrepancy between our findings and previous studies [30]. However, we note that the previous studies used two-copy females prepared by homozygosing individual transgenic lines, and so either the site of transgene insertion or mutations elsewhere on the homozygosed chromosome might have been responsible for the observed phenotypes.

### Nos- and Pum-dependent regulation of *bcd* mRNA in the testis

If the NRE plays no role in regulation of maternal *bcd* mRNA in the embryo, why then is it evolutionarily conserved [30]? One possibility is that the NRE mediates regulation of *bcd* during other stages of the life cycle. Although *bcd* mutants exhibit a classic “pure” maternal effect on embryonic development, we considered the possibility that low-level *bcd* transcription expression outside the ovary might generate mRNA that would be susceptible to NRE-mediated regulation by Pum and Nos. Indeed, RNAseq experiments reveal that *bcd* mRNA is expressed at low levels in a number of other tissues [35]. We chose to investigate *bcd* expression in males, thereby eliminating the possibility of sample contamination from the very high level of expression in ovaries.

**Fig 6:**
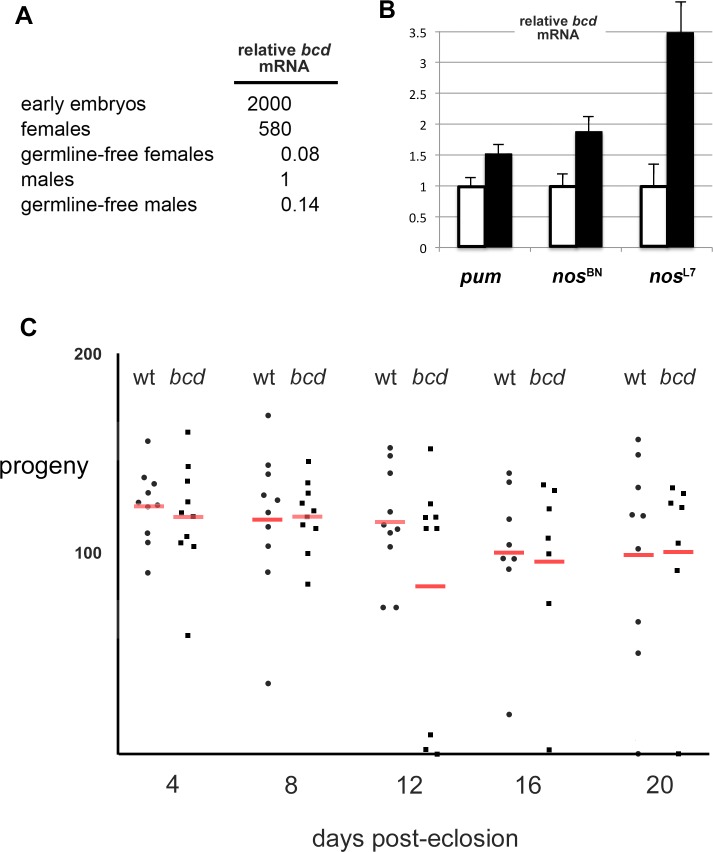
Pum- and Nos-mediated regulation of *bcd* mRNA in males. (A) Relative levels of *bcd* mRNA in various samples, normalized to the housekeeping mRNA for ribosomal protein S2 (Rps2) and with the level in males set to 1. Values are the average of two independent experiments. Germline-free animals were second-generation escapers from *tudor* mutant females, as described in the text. (B) The level of *bcd* mRNA in various mutant males (black bars, mutant genotypes below), relative to the level in wt males (white bars, set to a value of 1.0). Full mutant genotypes are: *pum*^Msc^ / *pum*^ET3^, *nos*^BN^ / *Df*, *nos*^L7^ / *Df*. The figure reports the value of the mean and the S.D. for four independent samples from qPCR experiments, measuring *Rps2* mRNA to calculate ΔCt. By the two-tailed t-test, each mutant is significantly different from the wt control (P ≤ 0.0047). (C) The fertility of individual wild type (wt) and *bcd* ^6^ / *bcd* ^12^ null mutant males is shown in serial matings performed from 4–20 days post-eclosion. Red bars report the average for each genotype; by two-tailed t-test, there is no significant difference between the two genotypes at any time point; the smallest value of P is 0.16 at day 12. Note that the average number of progeny on day 12 from *bcd* mutant males is somewhat artificially depressed by two moribund animals that died shortly thereafter.

In qPCR experiments, *bcd* mRNA is present in males, albeit at trace levels—in two independent biological replicates, we found that the level of *bcd* mRNA in males is 2000-fold lower than the maternally synthesized mRNA in 0–3 hr embryos (normalized to the level of a ribosomal protein encoding mRNA, Fig 6A). Perhaps not surprisingly given the expression level, we were unable to detect *bcd* mRNA by standard in situ methods in fixed testes or *bcd* protein either in situ or by Western blot of testes samples (not shown). However, we determined that most of the male *bcd* mRNA is expressed in the germ line, as follows. The majority of embryos from *tudor* (*tud*) mutant females develop severe abdominal segmentation defects and die; viable escapers that develop to adulthood lack germ line and are agametic [37]. The level of *bcd* mRNA in such germ line-free males is approximately seven-fold lower than the level in wild type males; the amount of residual mRNA in germ line-free males is only twofold above the level of detection. As a control, the level of *bcd* mRNA in escaper females is also reduced to a trace, close to the level of detection (Fig 6A).

Although Pum and Nos are thought to primarily regulate mRNA translation, they also destabilize some mRNA targets [38–40], including *hb* and *bcd* [29, 41]. Therefore, we asked whether Pum and Nos regulate the level of *bcd* mRNA in the testes.

We first attempted to measure the level of *bcd* mRNA in males with null alleles of *pum* and *nos* (*pum* ^MSC^ / *Df* and *nos*^RC^ / *Df*, respectively). However, qPCR measurements of *bcd* mRNA in these males were highly variable, perhaps because both genotypes are sub-viable and unhealthy. We therefore prepared relatively healthy males bearing the hypomorphic alleles *pum* ^*MSC*^ / *pum* ^*ET3*^ and *nos*^BN^ / *Df*. The activity of these alleles has been described elsewhere [20, 42, 43]. The *nos*^BN^ allele is due to a P-element insertion in the promoter that reduces transcription to different extents at different stages of development. We measured the level of *nos*^BN^ mRNA (that encodes wild type protein) and find considerable residual expression in males—41% of the wild type level (p<0.001).

As shown in Fig 6B, *bcd* mRNA is elevated in the *pum* and *nos* hypomorphic males described above 1.5-fold and 1.9-fold, respectively. The level of *nos* and *pum* expression in testes is significantly lower than in early embryos or ovaries [35]; we therefore imagine that the modest reduction in *pum* and *nos* activity in these hypomorphic backgrounds is sufficient to partially relieve regulation of *bcd* mRNA. These flies are relatively free of the pleiotropy associated with stronger loss-of-functions alleles, which is consistent with the idea that Pum and Nos normally act directly to destabilize *bcd* mRNA in males.

As a further test of the role of Nos, we examined *bcd* mRNA levels in the *nos* ^L7^ / Df(*nos*) mutant, which is selectively defective in repressing *hb* and *bcd* mRNAs [44]. The Nos^L7^ mutant protein is stable in vivo and bears a 7-amino acid deletion in its carboxy-terminal tail, which is essential for mediating cooperative binding with Pum to the *hb* and *bcd* NREs in vitro [20, 21]. In vivo, the Nos^L7^ mutant protein does not repress *hb* and *bcd* mRNAs in the embryo, although it retains the ability to perform other *nos*-dependent activities, for example during oogenesis [44]. We find that *bcd* mRNA levels are elevated 3.5-fold in *nos* ^L7^ / Df(*nos*) males (Fig 6B), further supporting the idea that Pum and Nos act directly by binding to the *bcd* NRE to destabilize the mRNA in testes. The resulting elevation of *bcd* mRNA has no apparent functional significance, since the *nos* and *pum* mutant males used in the experiments of Fig 6B have morphologically normal testes and exhibit normal fertility. Moreover, we have been unable to identify a role for *bcd* itself in males. The testes of *bcd* mutant males are morphologically normal (not shown), and the males exhibit normal fertility for at least 20 days post-eclosion (Fig 6C), significantly longer than the 14 day half-life of male germ line stem cells [45]. In conclusion, while Pum and Nos appear to regulate the steady state level of *bcd* mRNA in the testis, the biological significance of this regulation is currently unclear.

## Discussion

The NRE was originally defined as a functional unit conferring Nos-dependent regulation in vivo [25]. In the *hb* 3’-UTR, two NREs are necessary and sufficient (if inserted in another maternal “reporter” mRNA) to mediate translational repression and mRNA destabilization [25]. Subsequent work showed the *hb* NRE to be a scaffold that, in addition to binding Nos, binds one high-specificity, high-affinity protein (Pum) [46–48] and another relatively low-specificity, low-affinity protein (Brain Tumor) [49]. On its own, Nos does not bind specifically to the NRE, but does so jointly with Pum [20]; the structural basis for this cooperative binding has recently been elucidated [21]. In Pum or Nos mutants, *hb* is not detectably repressed [41, 50]; in Brat mutants, *hb* is weakly repressed (perhaps because only partial loss-of-function alleles can be analyzed) [51]. Finally, cis-acting mutations in the Pum-, Nos-, and Brat-binding sites reduce or eliminate NRE function in the embryo [24, 47]. Taken together, the evidence definitively shows that Pum recruits Nos to the NRE, and that both factors (and Brat) are essential for regulation of *hb* mRNA. No other Nos regulatory target has been as thoroughly studied.

The 45 nucleotide NRE in *bcd* was also defined functionally as a sequence necessary for repression of the mRNA when exposed to Nos; the clearest manifestation of *bcd* NRE activity is in embryos with ectopic anterior Nos [25, 28, 29]. Two *bcd* NREs confer partial repression when substituted for the *hb* NREs [25], suggesting both elements assemble similar repressor complexes. We show in Fig 3 that Pum binds to the *bcd* NRE and that mutations that eliminate binding of Pum abolish Nos-dependent repression in vivo. Goldstrohm and colleagues have recently shown that Pum and Nos bind cooperatively to a 16-nucleotide fragment of the *bcd* NRE [21]. Taken together, these observations are consistent with a model in which Pum recruits Nos to the *bcd* NRE to repress translation.

Do Pum or Pum-related proteins regulate mRNAs in vivo in the absence of Nos? In the case of the budding yeast Puf proteins, this is certainly the case, since no yeast ortholog of Nos is thought to exist. Instead of depending on Nos and its ability to recruit various effectors [19, 52, 53], the yeast Puf proteins are thought to interact directly with translational repressors. The best characterized of these interactions is with the Pop2 subunit of a multiprotein deadenylase complex that inhibits translation and promotes mRNA degradation [27]. Thus, it appears that yeast Puf proteins act on their own to recruit inhibitors to their mRNA regulatory targets.

In Drosophila, eliminating function of *pum* or *nos* has different effects in the peripheral nervous system [16] and at the neuro-muscular junction [14], suggesting that at least some target mRNAs in these cell types are regulated by Pum independent of Nos. In the early embryo, two lines of evidence have been cited to support Nos-independent regulation by Pum. The first of these is from Gamberi et al. (2002), whose observations we have been unable to reproduce (as described above). The second line of evidence emerged from studies of the PanGu (PNG) kinase in early embryonic development. PNG is required for normal accumulation of Cyclins B and A throughout the prospective somatic cytoplasm of the embryo. Vardy and Orr-Weaver found a strong genetic interaction, in which loss of *pum* function restores levels of both Cyclins in embryos that lack PNG activity [54, 55]. This suppression is effective throughout the embryo, even at the anterior, and thus is presumably Nos-independent. However, cis-acting sites that mediate repression by Pum in the somatic cytoplasm have not yet been defined in either *Cyc* mRNA; moreover, no significant binding of Pum to either *Cyc* mRNA was detected in a survey of the embryonic transcriptome [39]. It has become clear that Pum and PNG regulate (in aggregate) thousands of mRNAs in the embryo [38, 39, 56, 57], raising the possibility that they act indirectly to control CycB and CycA protein accumulation. In summary, Nos-independent regulation by Pum is likely to occur during other stages of development in Drosophila, but not in the syncytial cleavage stage embryo.

In artificial contexts Pum and Nos have been shown to act independently of each other. For example, in experiments using transfected Drosophila cell lines, Pum can repress NRE-bearing reporter mRNAs in the absence of Nos [21]. And tethering of Nos with exogenous RNA-binding proteins can impose repression on suitably engineered reporter mRNAs both in S2 cells and in the early embryo [19, 52]. While these experiments have been useful in dissecting mechanism, they do not fundamentally change the interpretation of in vivo experiments, in which Pum, Nos, and Brat are each required for repression of *hb* via native regulatory signals, for example. Macdonald and co-workers have shown that reporter mRNAs bearing minimal binding sites for the translational repressor Bruno can be used to monitor regulation in the ovary [58]; to our knowledge, no similar experiments to test the function of minimal Pum binding sites in vivo have been reported.

While we find no significant regulation of *bcd* mRNA by Pum or Nos in the embryo, they do appear to destabilize the low level of *bcd* mRNA present in testes. However, we have been unable to detect an obvious function for *bcd* itself in the male germline (Fig 6C). Perhaps *bcd* plays a subtle role in males not evident in the laboratory; alternatively, regulation of *bcd* by Nos in the testis may be gratuitous, with the NRE maintained under selective pressure due to regulation of the low levels of *bcd* present in some other cell type.

Similarly, there are no apparent consequences of relieving Pum- and Nos-dependent regulation of *bcd* mRNA in the testis; and it is unclear whether such regulation is direct or not. Our strongest argument that Pum and Nos act directly on the *bcd* mRNA in testes comes from the stabilization of *bcd* mRNA in *nos* ^L7^ mutant flies that encode a mutant protein specifically defective in cooperative binding with Pum to the *hb* and *bcd* NREs [20, 21]. However, Pum and Nos likely have many regulatory targets and thus could control *bcd* mRNA levels indirectly. If the regulation is direct, then the level of *bcd* NRE(mut) mRNA should be the same in wild type and *nos* mutant males. However, we are unable to test this idea with currently available reagents; our *bcd* transgenes are faithfully expressed in the ovary but wildly over-expressed in males (on average 50-fold), with tremendous variation from line to line (4.5- to 150-fold, see Methods for details). One possible explanation of the variable over-expression is that the *bcd* promoter is weak in males and thus susceptible to chromosomal position-effects of transgene insertion on its activity. In any case, while we favor the idea that Pum and Nos act directly on *bcd* mRNA in the testes, definitive evidence is lacking.

In summary, while several cases of Nos-independent regulation by Pum in Drosophila have been reported [14, 16], many other Pum mRNA targets appear to be regulated jointly with Nos (for example, see also [15, 17, 59]). Perhaps joint regulation by Pum and Nos is more efficient than by Pum alone. Rapid developmental decisions (such as repression of *hb* mRNA in the syncytial cleavage stage embryo) may demand joint action of both factors, whereas Pum alone may suffice during the more leisurely remodeling of the neuro-muscular junction, which occurs over the course of several days.

## Materials and methods

### *Drosophila* strains

Flies were grown at 25º and crossed to generate genotypes used in various experiments as follows: *pum* ^*MSC*^ / *pum*^*FC8*^ (Fig 1A–1C), *pum* ^*MSC*^ / Df *pum*^*BSC24*^ (Bloomington stock #6756) (Fig 1D), and *pum* ^*MSC*^ / *pum* ^*ET3*^ (Fig 6B); *nos*^*BN*^ (Fig 2), *nos*^*BN*^ / Df *nos* ^Exel6183^ (Bloomington stock #7662) (Fig 6B), *nos*^*L7*^ / Df *nos* ^Exel6183^ (Fig 6B); *bcd* ^6^ / *bcd 12* (Fig 6C); *tud* ^1^ / Df *tud* ^Exel6072^ (Bloomington stock #7554) (Fig 6A); wild type, *w* ^1118^. Note that the *nos*^BN^ allele has significant residual function in some tissues and developmental time points (e.g., early oogenesis, males) but that neither *nos* mRNA nor protein is detectable in embryos from *nos*^BN^ females [43]. The *bcd*^6^ and *bcd*^12^ alleles have stop codons and give rise to no detectable protein in embryos [2]. Flies bearing a *nos*^+^—[*bcd* 3’-UTR] transgene essentially identical to one described [60] were prepared by germline transformation. Fertility assays of Fig 6C were performed by selecting newly eclosed wild type (*w*^1118^) and *bcd* ^6^ / *bcd* ^12^ males (day 0), and mating them individually to 2 new virgin wild type females every fourth day. Mated females were returned to the appropriate vial and allowed to lay eggs until the next generation of flies was about to emerge, when the mated females were discarded. After incubating the vials for a further 5 days (to allow fertilized eggs to pupariate, pupae and empty pupal cases were counted to measure fecundity.

### RNA in situ hybridization and immunohistochemistry

*bcd* and *eve* mRNAs were detected by standard in situ hybridization methods using digoxigenin-labeled cDNA probes and visualized with a Zeiss Axiophot microscope using Nomarski optics and a Q Imaging digital camera. For immunohistochemical experiments, embryos were fixed in 4% formaldehyde and incubated with rabbit anti-Bcd sc-66818 (at 1:100, Santa Cruz Biotechnology) and the 2B8 anti-grasshopper Eve (at 1:50) developed by Kai Zinn obtained from the Developmental Studies Hybridoma Bank, developed under the auspices of the NICHD and maintained by The University of Iowa. Secondary antibodies (1:200) were from Jackson ImmunoResearch Laboratories. Embryos were visualized on a Zeiss 510 confocal microscope. The timing of embryonic development was assessed both by morphology [61] and evolution of the *eve* mRNA pattern [31], with Bownes stages as described [61]. An approximate timeline for early embryonic development is: 0–1 hrs, stages 1–2 (fertilization-preblastoderm); 1–2 hrs, mid-stage 2–4 (pre- and syncytial blastoderm); 2–3 hrs, stage 5 (cellularization); 3–4 hrs, stage 6–8 (gastrulation-germ band elongation); 4–5 hrs, stage 9–10 (germ band elongation- gnathal lobe formation).

### *bcd* transgenes

We used a 9 kb fragment of *bcd* ^+^ genomic DNA that includes the entire transcription unit, 4 kb of 5’-flanking DNA (and thus a portion of the adjacent *Ama* gene), 1 kb of 3’-flanking DNA, and that fully rescues the maternal *bcd* phenotype, as described [62]. The fragment was modified by addition of a NheI site immediately downstream of the translation termination codon. DNA encoding the NRE (mut1), and NRE (Δ) mutations was inserted in separate reactions between the NheI site and the endogenous MluI site; modified *bcd* constructs were inserted into pCasper4 and germline transformants generated by standard methods. The DNA Strider program was used to analyze sequences [63].

### Molecular biology experiments

Electromobility gel shift experiments were performed essentially as described [19] using RNA prepared by transcription of derivatives of T4425 bearing SpeI-BamHI inserts that encode the NRE sequences of Fig 2. For Western blots, embryos were prepared and sorted essentially as described [34]. Sorted embryos pools were homogenized in sample buffer without BPB and the concentration of protein in each lysate determined by Bradford assay. Lysates were electrophoresed through 8% SDS-containing gels, transferred to nitrocellulose and incubated first with anti-Bcd sc-66818 (1:1000) followed by HRP-coupled goat anti-rabbit (Jackson ImmunoResearch) at 1:7500. For detection of alpha-Tubulin, monoclonal antibody (Sigma T5168) was incubated at 1:10000 followed by HRP-coupled secondary antibody at 1:5000. Blots were visualized with ECL-plus (Amersham). Total RNA was extracted from embryo collections by homogenization in Trizol (Ambion). Preparation of cDNA and qPCR reactions were performed according to the manufacturer (Applied Biosystems), measuring the levels of *bcd* (assay DM02148187_g1) or *nos* mRNAs (DM02134535_g1) relative to the level of *Rps2* mRNA (DM02361142_s1). Measurement of the relative level of *bcd* mRNA was assayed in three independent samples prepared from pools of 50 males for the experiment of Fig 6B. Measurement of the relative level of *nos* mRNA in *nos*^BN^ / Df males referred to in the text was assayed in four independent samples from pools of 50 males. In each case statistical significance was determined using an unpaired t test. To measure the level of *bcd* transgene-encoded mRNA, we collected duplicate samples of 0–3 hour embryos from females bearing a single transgene copy and duplicate samples of single-copy transgenic males. Four *bcd* (NRE)+ transgenes and four *bcd* (NRE)mut transgenes were analyzed. The average level of *bcd* mRNA in the 8 embryonic samples was 1.6-fold higher than in wild type, close to the expected value of 1.5; but the average level in the 8 male samples was 76-fold higher than in wild type.
